# Comparative Proteomic Analysis of Soybean Leaves and Roots by iTRAQ Provides Insights into Response Mechanisms to Short-Term Salt Stress

**DOI:** 10.3389/fpls.2016.00573

**Published:** 2016-04-29

**Authors:** Wei Ji, Ru Cong, Sheng Li, Rui Li, Zhiwei Qin, Yanjun Li, Xiaolin Zhou, Sixue Chen, Jing Li

**Affiliations:** ^1^Department of Plant Biotechnology, College of Life Science, Northeast Agricultural UniversityHarbin, China; ^2^Department of Vegetables, College of Horticulture, Northeast Agricultural UniversityHarbin, China; ^3^Department of Biology, Genetics Institute, University of FloridaGainesville, FL, USA; ^4^Department of Proteomics, Interdisciplinary Center for Biotechnology Research, University of FloridaGainesville, FL, USA

**Keywords:** soybean, NaCl, leaf, root, quantitative proteomics, iTRAQ

## Abstract

Salinity severely threatens land use capability and crop yields worldwide. Understanding the mechanisms that protect soybeans from salt stress will help in the development of salt-stress tolerant leguminous plants. Here we initially analyzed the changes in malondialdehyde levels, the activities of superoxide dismutase and peroxidases, chlorophyll content, and Na^+^/K^+^ ratios in leaves and roots from soybean seedlings treated with 200 mM NaCl at different time points. We found that the 200 mM NaCl treated for 12 h was optimal for undertaking a proteomic analysis on soybean seedlings. An iTRAQ-based proteomic approach was used to investigate the proteomes of soybean leaves and roots under salt treatment. These data are available via ProteomeXchange with the identifier PXD002851. In total, 278 and 440 proteins with significantly altered abundances were identified in leaves and roots of soybean, respectively. From these data, a total of 50 proteins were identified in the both tissues. These differentially expressed proteins (DEPs) were from 13 biological processes. Moreover, protein-protein interaction analysis revealed that proteins involved in metabolism, carbohydrate and energy metabolism, protein synthesis and redox homeostasis could be assigned to four high salt stress response networks. Furthermore, semi-quantitative RT-PCR analysis revealed that some of the proteins, such as a 14-3-3, MMK2, PP1, TRX-h, were also regulated by salt stress at the level of transcription. These results indicated that effective regulatory protein expression related to signaling, membrane and transport, stress defense and metabolism all played important roles in the short-term salt response of soybean seedlings.

## Introduction

Salinity is one of the most widespread agricultural problems in arid and semi-arid regions and significantly reduces plant growth and productivity. It is reported that 20% of irrigated land, which yields one-third of the world's food, is threatened by salt stress (Ma et al., 2012). High salinity, predominantly in the form of NaCl, affects plant growth mainly in three ways: osmotic stress, ion toxicity and secondary stresses such as oxidative stress (Turkan and Demiral, 2009). The salt signal is primarily perceived through roots, which rapidly respond to maintain root functionality and transmit signals to other organs for appropriate response and adaptation in the entire plant (Zhao et al., 2013). A combinatorial approach of accelerated gene discovery through genomics, proteomics, and advances in plant biotechnology techniques will provide insights into the molecular and biochemical basis of plant stress tolerance, which ultimately lead to crop improvement for sustainable agriculture (Eldakak et al., 2013).

Soybean (Glycine max) is one of the most economically important crops due to its high content of seed oil and protein. Salt stress affects soybean plant growth throughout its development starting from seed germination to flowering, however the early vegetative growth stages are reported to be more prone to abiotic stresses (Hossain et al., 2013). Recently, with advances in transcriptome mapping, some salt-responsive genes and molecular regulatory pathways have been identified in soybean seedlings (Fan et al., 2013; Qi et al., 2014). However, genomic studies only highlight mRNA levels, which may not be necessarily translated into proteins, and therefore transcriptome data may not correlate with results from proteomic analysis due to post-transcriptional and post-translational modifications (Hossain et al., 2013). Thus, quantitative analysis of gene expression at the protein level is essential for determining plant responses to salt stress. The proteome of soybean subjected to salinity has been analyzed using roots and hypocotyls of young seedlings (Aghaei et al., 2009) and other tissues (Sobhanian et al., 2010), and indicated that photosynthesis, protein biosynthesis and ATP biosynthesis were decreased while defense protein increased in soybean in response to salt stress (Sobhanian et al., 2011). Further comparative proteomic approaches have been employed to explore proteome expression patterns in germinating soybeans under salt stress treatments, the results suggested that enhanced energy metabolism and accelerated protein processing in the endoplasmic reticulum were important strategies for germinating soybeans response to NaCl stress (Yin Y. Q. et al., 2014). In addition, a proteomic approach has also been applied to seedlings of different salt tolerant genotypes of soybean under salt stress (Ma et al., 2012, 2014), which identified several proteins as potential candidates for augmenting salt tolerance in soybean. However, the determination of the mechanisms involved in salt tolerance remains a challenging task; because plant responses to salinity can be very diverse, depending on the severity and duration of the stress, this leads to various changes at the proteome level (Hossain et al., 2013). The initial phases of stress response usually reveal more profound differences in the composition of the proteome compared to later phases of stress since novel homeostasis between plant and environment has been established (Vitamvas et al., 2015). The majority of studies that investigated salt response in soybean have focused on relatively late response to salinity treatment, in contrast, the early response of plants to short-term salt stress has been overlooked (Liu et al., 2014; Pi et al., 2016). Therefore, the main objectives of this study were to investigate the proteome expression patterns and to identify the differentially expressed proteins under short-term salt stress in soybean seedlings. In the present study, an iTRAQ-based proteomic technique was used to assess proteome changes and identify proteins that were differentially expressed in soybean leaves and roots in response to 12 h of 200 mM NaCl treatment. Our approach was sensitive enough to identify 278 and 440 proteins with significantly altered abundance in leaves and roots of soybean, respectively. Proteins with markedly altered expression patterns were classified into 13 functional groups. Candidate proteins that may play important roles in salt stress responses were analyzed at the transcript level via semi-quantitative RT-PCR. This study advanced our understanding of salt-responsive mechanisms in soybean plants.

## Materials and methods

### Plant materials and salt treatment

Seeds of soybean (*Glycine max* cv Dongnong 50) were germinated on filter paper soaked in distilled water in Petri dishes at 25°C. After 2 days, uniform germinated seedlings were transferred to plastic containers filled with vermiculite and irrigated with 1/4 Hoagland nutrient solution (Hoagland, 1944) in a growth chamber under normal conditions (25/20°C day/night temperature, relative humidity of 60–80% and 16 h light period/day at intensity of 160 μmol photons m^−2^ s^−1^). When the plants reached the trefoil stage, soybean plants were transferred to liquid medium containing 1/4 Hoagland nutrient. For stress treatment, half of the soybean plants were shifted to 1/4 Hogland solution containing 200 mM NaCl for 0, 1, 3, 6, 12, 24, 48 h. The rest of the seedlings, grown in liquid 1/4 Hogland solution with no NaCl added were used as controls. Plant roots, and the second developed trifoliate leaves were analyzed at the proteomic, physiological and transcript levels. Three independent sets of control and NaCl treated samples were collected, and each replicate represented a pooled sample of three individual plants.

### Measurement of superoxide dismutase activity, peroxidase activity, malonyldialdehyde, and chlorophyll

Leaf and root samples (0.4 g) was ground in liquid nitrogen and homogenized in 10 volumes of ice-colded 50 mM sodium phosphate buffer (pH 7.8). After centrifugation at 15,000 g at 4°C for 20 min, the resulting supernatants were collected and used for protein content assay and enzyme activities. Protein content was determined according to Bradford (Bradford, 1976) with bovine serum albumin as the standard. Superoxide dismutase (SOD) activity was determined by monitoring its ability to inhibit photochemical reduction of nitroblue tetrazolium (NBT) at 560 nm (Beauchamp and Fridovich, 1971). The activity of peroxidase (POD) was determined using the guaiacol oxidation method (Bradford, 1976). Malondialdehyde (MDA) content was measured by the thiobarbituric acid (TBA) reaction according to the method of (Hodges et al., 1999). MDA contents were calculated from UV absorbance at 450, 532, and 600 nm. Leaf chlorophyll was extracted in 80% acetone and measured with a UV–visible spectrophotometer at 645 and 663 nm. Chlorophyll a, chlorophyll b and total chlorophyll contents were calculated according to the formular previously described (Arnon, 1949).

### Measurement of Na^+^/K^+^ contents

Dried roots and leaves of soybean seedlings were used for analysis of Na^+^ and K^+^ contents. The samples were ground to a powder using a pestle and mortar. A portion of the powdered samples were digested with concentrated HNO_3_ at 110°C for 2 h. Na^+^ and K^+^ contents in the digested samples were measured using an atomic absorption spectrophotometer as described previously (Wang and Zhao, 1995).

### Protein extraction and quantification

Total protein from three biological replicates were prepared from control and NaCl-treated soybean leaf tissues using a phenol extraction method (Wang L. et al., 2014) with the following modifications. Briefly, 1 g of each sample were ground into fine powder in liquid nitrogen in a chilled mortar. After adding 2.5 mL of Tris pH8.8 buffered phenol and 2.5 mL of extraction buffer (0.1 M Tris-HCl pH 8.8, 10 mM EDTA, 0.4% β-mercaptoethanol, 0.9 M sucrose), the samples were homogenized for 15 min, then transferred to a 50 mL tubes and agitated for 30 min at 4°C, followed by centrifugation at 10,000 × g for 30 min at 4°C. The phenol phase was removed to new tubes, and the rest pf the aqueous phase was back-extracted with 4 mL extraction buffer and 4 mL phenol. The two extractions were combined and precipitated by adding 5 volumes of 0.1 M ammonium acetate in 100% methanol and incubating at −20°C overnight. The precipitate was collected by centrifugation at 20,000 × g for 20 min at 4°C, and washed twice with 0.1 M ammonium acetate in methanol, ice-cold 80% acetone, and once with cold 70% ethanol. The resulting pellets were dissolved in lysis buffer (7 M urea, 2 M thiourea, 4% Chaps, 40 mM DTT). Protein concentrations were determined using Bradford assay (Bio-Rad) using BSA as the standard.

### Protein digestion, iTRAQ labeling and strong cation exchange fractionation

A total of 100 μg of protein from each sample was used for acetone precipitation overnight. After protein precipitation, the pellet of each replicate was dissolved in 1% SDS, 100 mM triethylammonium bicarbonate, pH 8.5. The samples were reduced, alkylated, and digested with trypsin at 20:1 (w/w) at 37°C for 12 h, then labeled using the iTRAQ Reagents 8-plex kit according to the manufacturer's instructions (AB Sciex Inc., USA). The untreated leave and root sample replicates were labeled with iTRAQ tags 113, 117, and the salt-treated samples labeled with tags 115, 119, respectively. Three sets of iTRAQ samples were used for the three biological replicates. After labeling, the samples were combined and lyophilized. The peptide mixture was dissolved in strong cation exchange (SCX) solvent A (25% (v/v) acetonitrile, 10 mM ammonium formate, and 0.1% formic acid, pH 2.8), and then fractionated on an Agilent HPLC system 1260 with a polysulfethyl A column (2.1 × 100 mm, 5 μm, 300 Å). Peptides were eluted at a flow rate of 200 μL min^−1^ with a linear gradient of 0−20% solvent B (25% v/v acetonitrile, 500 mM ammonium formate, pH 6.8) over 50 min, followed by ramping up to 100% solvent B in 5 min and holding for 10 min. The absorbance at 280 nm was monitored and a total of 12 fractions were collected.

### Reverse phase nanoflow HPLC and tandem mass spectrometry

Each SCX fraction was lyophilized and dissolved in solvent A (3% v/v acetonitrile, 0.1% v/v acetic acid), and loaded onto a C18 PepMap nanoflow column (75 μm internal diameter, 3 μm, 100 Å). Peptides from iTRAQ samples were separated using a 90 min linear gradient ranging from 97% solvent A/3% solvent B (96.9% v/v acetonitrile, 0.1% v/v acetic acid) to 40% solvent A/60% solvent B. MS/MS analysis was carried out on a LTQ Orbitrap Elite mass spectrometer (Thermo Scientific, Bremen, Germany) in a positive mode (Parker et al., 2015). Briefly, full MS survey scan was performed from a mass range of 400–1800 m/z with resolution *R* = 60,000 at m/z 400. HCD fragmentation was used for MS/MS, and the 10 most intense signals in the survey scan were fragmented. A resolution of 7500 at 400 m/z was used with an isolation width of 1 m/z, 30,000 signal threshold.

### Data analysis and interpretation

The raw MS/MS data files acquired from the Orbitrap were processed by a thorough database searching considering biological modification and amino acid substitution against the Uniprot Soybean database with 71,042 entries (downloaded on May 16, 2013), using Proteome Discoverer 1.4 (Thermo Scientific Inc., Bremen, Germany) with the SEQUEST algorithm. The following parameters were used for searching: lowest and highest charge: +2 and +5, respectively; minimum and maximum precursor mass: 300 and 6000 Da, respectively; minimum S/N ratio: 3; enzyme: trypsin; maximum missed cleavages: 1; FDR ≦ 0.01; mass tolerance: 10 ppm for precursor ions and 0.5 Da for fragment ions; dynamic modifications: phosphorylation (+79.966 (S,T,Y)), carbamidomethyl (+57.021 Da (C)), oxidation (+15.995 Da (M)), carbamidomethyl (+57.021 Da (C)). The N-terminal modification was set for iTRAQ8plex (+304.205 Da). The Proteome Discoverer results files (.msf) were uploaded to ProteoIQ 2.6 (NuSep) software for further filtering. Peptide probability was applied to filter peptide assignments obtained from MS/MS database searching results using predictable false identification error rate. Protein probability was used to filter proteins with the null hypothesis that the database matching is random and taking into account of the peptide probability for all the peptides apportioned to that protein (Koh et al., 2015). Proteins detected with at least three spectral counts, FDR ≤ 5%, 95% probability and listed as top scoring proteins are considered as high confidence matches and are presented in the results. To be identified as being significantly differentially expressed, a protein should be quantified with at least three peptides in each experimental replicate, a *p*-value smaller than 0.05 and fold change greater than 1.3 or less than 0.7. The MS proteomics data have been deposited in the ProteomeXchange Consortium via the PRIDE partner repository (Vizcaino et al., 2013) with the data set identifier PXD002851. The functional annotation of proteins found was determined by Blast2GO (Bioinformatics Department, CIPF, Valencia, Spain), and then grouped on the basis of their biological functions from Gene Ontology (GO) terms combined with information from the literature (Zhang et al., 2012; Zhao et al., 2013). The protein-protein interaction network was constructed using the String program (http://string-db.org).

### RNA extraction and semi-quantitative RT-PCR

Total RNA was extracted from salt-treated and control soybean leaves and roots separately by Ultrapure RNA Kit (Beijing Comwin Biotech Co., Ltd., China) with DNase I treatment, and cDNA was reverse transcribed from 1 μg of total RNA using a First Strand cDNA Synthesis Kit (Invitrogen). Gene-specific primers (GSPs) used for RT-PCR were designed with the Primer 5 software according to soybean cDNA sequences (Table S1). The soybean actin 11 gene was used as endogenous control for normalization. The annealing temperatures and numbers of amplification cycles of these 6 genes in the PCR assay were shown in Table S1. PCR was run with a program consisting of a 95°C denaturation for 5 min, a 95°C denaturation for 30 s, followed by a 50–62°C annealing for 30 s, and a 72°C extension for 60 s over certain cycles by 2 × Es Taq MasterMix (Beijing Comwin Biotech Co., Ltd., China). The mRNA expression level was analyzed by PCR product with certain cycle number (26, 30, and 33) coupled with 1.5 % agarose gel electrophoresis.

## Results

### Physiological changes of soybean plants under salt treatment

**Figure 1:**
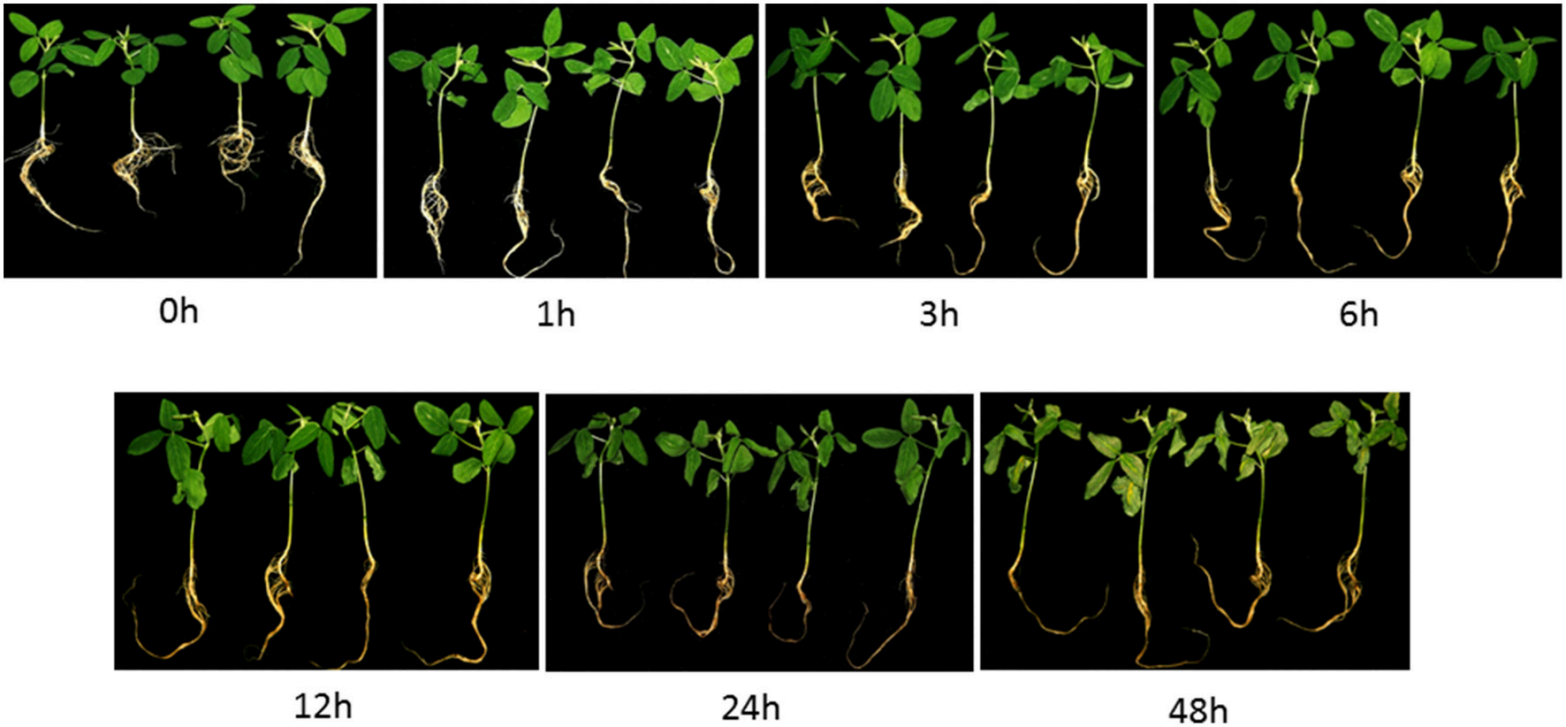
**Morphological changes of soybean seedlings after treatment with 200 mM NaCl for 0, 1, 3, 6, 12, 24 and 48 h**. The photographs showed similar plants at the indicated time points which were representative of three independent experiments.

The exposure of soybean seedlings to 200 mM NaCl resulted in various morphological and physiological changes in the leaves and roots over time. Salinity stress resulted in a clear growth retardation of plants. Although treatment with 200 mM NaCl for 1 h did not induce any obvious phenotypic differences in the seedling leaves. Treatment for 3 h induced the older leaf margins to roll inward (Figure 1), and this phenotype was quite obvious after treatment for 12 h. After 12 h, the leaves of salt-treated seedlings started to wilt, and chlorotic spots became visible (Figure 1). When treated for 48 h, the curling leaves displayed severe chlorosis and dried up.

**Figure 2:**
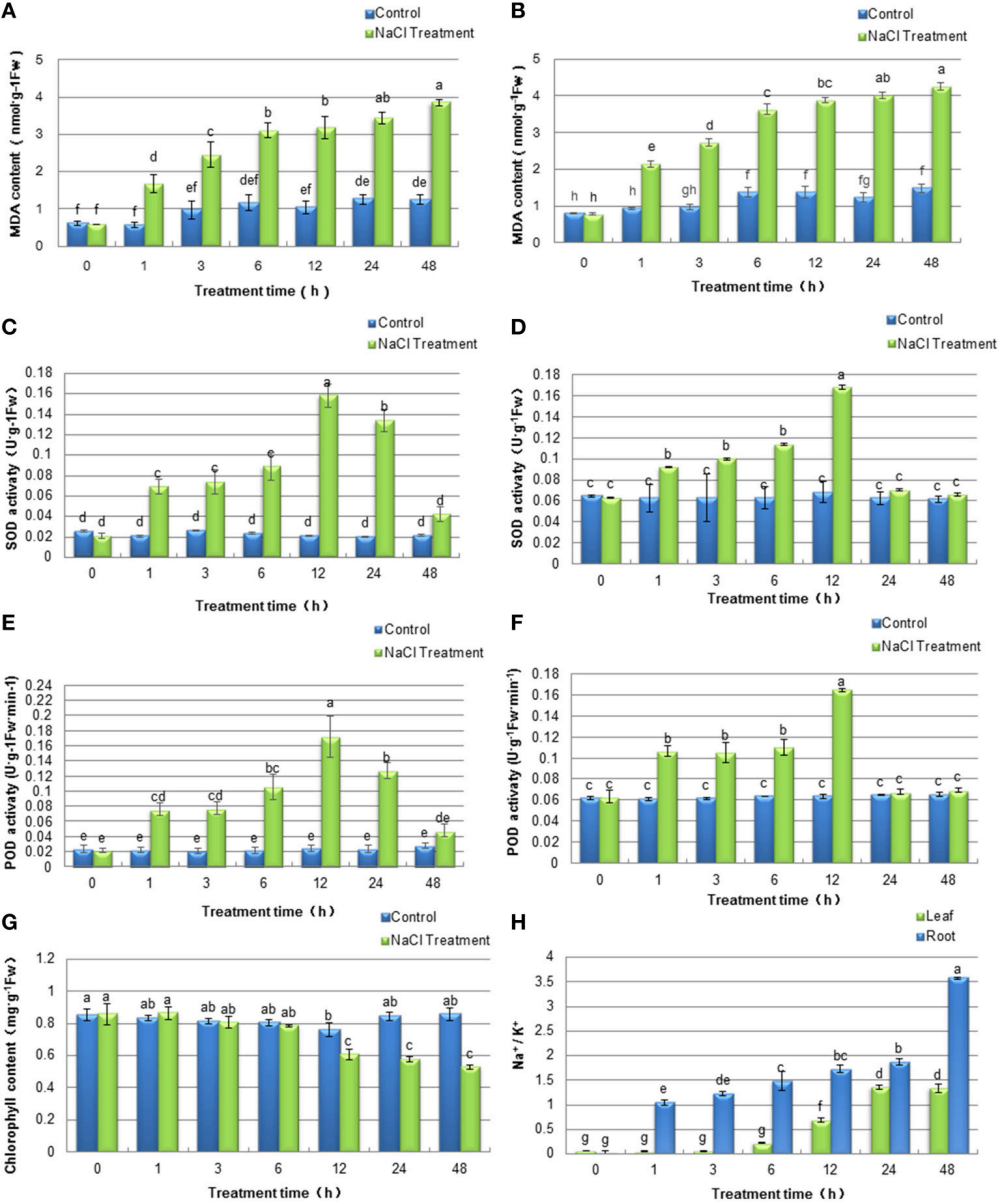
**Effects of salinity stress on physiological characteristics of soybean leaves and roots**. Soybean seedlings were treated with 200 mM NaCl for 0 (control), 1, 3, 6, 12, 24, 48 h. The values represented the mean ± SD of three biological replicates. The different letters above columns indicated significant differences among different treatment time of soybean plants based on one-way ANOVA (*p* < 0.05). **(A,B)** MDA contents of soybean leaves and roots respectively; **(C,D)** SOD activities of soybean leaves and roots respectively; **(E,F)** POD activities of soybean leaves and roots respectively; **(G)** Chlorophyll content of soybean leaves; **(H)** Na^+^/K^+^ ratios of soybean leaves and roots.

To evaluate the effects of stress on soybean seedlings during the 48 h of NaCl treatment and determine the time point for sample collection after salt stress for subsequent proteomics analysis, physiological experiments were conducted. Generally, the concentrations of MDA is one of major indicators of stress-triggered oxidative damage and reactive oxygen species (ROS) accumulation. To monitor the effects of salt treatment on the plasma membrane system in soybean plants, MDA contents in the leaves and the roots were detected. As shown in Figures 2A,B, the MDA levels increased during the time course of the experiment in both tissues, which indicated that the injury to plasma membrane systems accumulated over the length of the NaCl treatment. The antioxidant property in plant tissue is generally accepted to correlate with plant tolerance to salt stress and it is usually represented by general radical scavenging capacities of superoxide dismutase (SOD) and peroxidases (POD). In the present study, the activities of SOD (Figures 2C,D) and POD (Figures 2E,F) exhibited similar dynamic patterns at different time points both in soybean leaves and roots. After treatment, the activities of SOD and POD increased initially during the first 12 h, both of which peaked at 12 h and declined from this time point. The results indicated that the ROS scavenging capacities of soybean seedlings were at the highest after 12 h of salt treatment. Thus, we speculated that a defensive mechanism may have developed in soybean seedlings against salt stress at this time point. Consistent with the phenotype of soybean seedlings under salt treatment, total chlorophyll content in the soybean leaves was significantly decreased after 12 h of salt stress treatment (Figure 2G), which was similar to a previous study in rice (Xu et al., 2015). Furthermore, salt stress significantly affected the concentrations of Na^+^ and K^+^ in soybean leaves and roots (Figure 2H). The Na^+^/K^+^ ratios in soybean leaves and roots dramatically increased under salt stress, and the roots Na^+^/K^+^ ratios in soybean seedlings were significantly higher than that in leaves.

### iTRAQ analysis and identification of differentially expressed proteins

**Figure 3:**
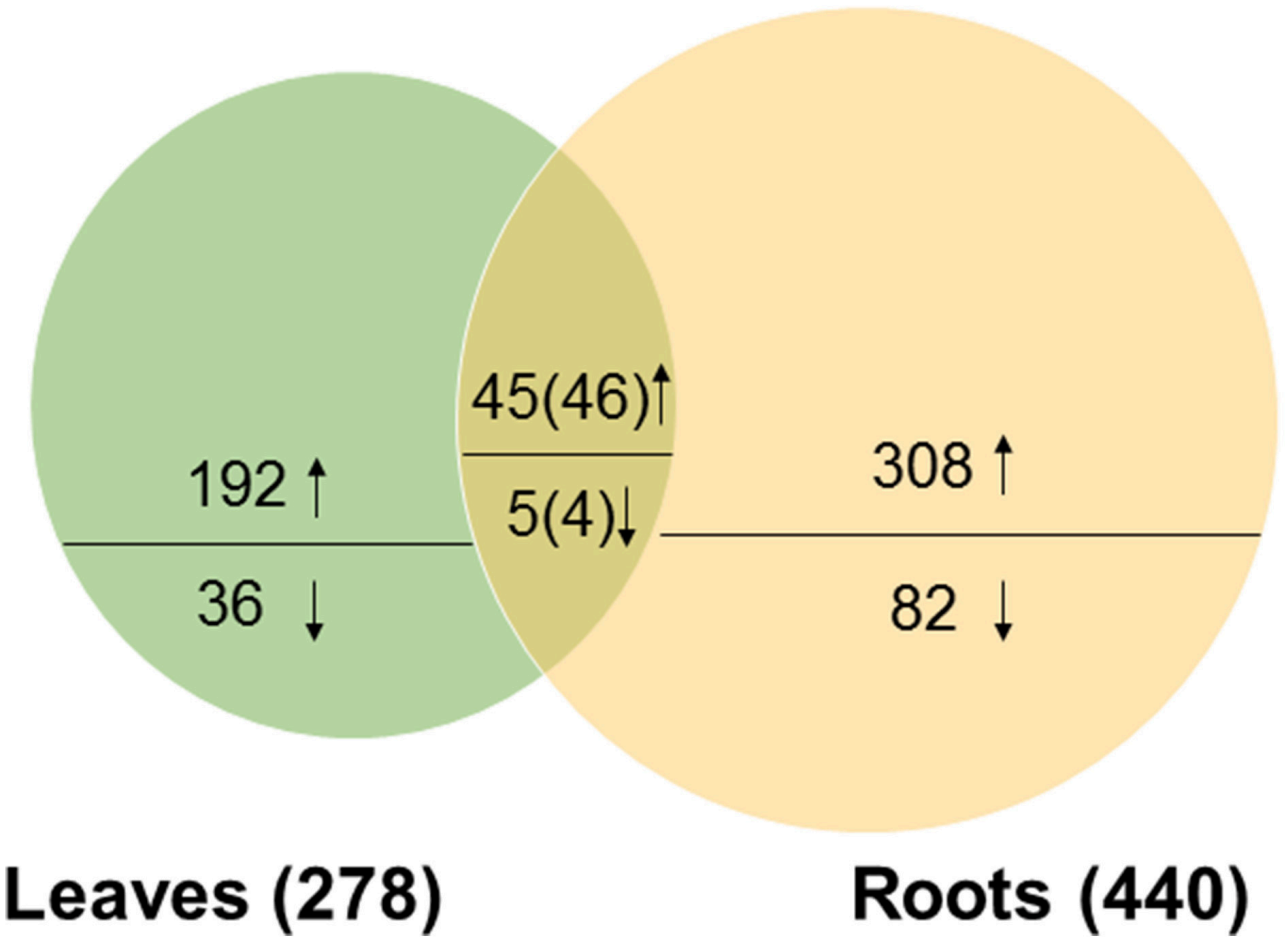
**Venn diagram of the distribution of differentially expressed proteins responsive to salt stress in soybean leaves and roots**. The number above or below the horizontal line in each portion indicated the number of up-regulated or down-regulated proteins. The overlapping regions indicated the number of common proteins. Among the 50 common DEPs, 45 were up-regulated and 5 were down-regulated in leaves; and 46 were up-regulated and 4 were down-regulated in roots.

Given the physiological characteristics of salt treated soybean seedlings, a time of 12 h with 200 mM NaCl treatment was deemed as optimal to explore early responses to salt stress in soybean. Consequently, changes in the leaf and root proteomes of soybean seedlings subjected to 200 mM NaCl for 12 h were analyzed using iTRAQ-LC/MS-MS. Data from three biological replicates were analyzed and proteins detected by querying data with a soybean protein database. In total, 142,714 spectra could be matched to the database, resulting in a total of 48,714 peptides which were assembled into 6610 non-redundant protein groups (Table S2). Differentially expressed proteins (DEPs) were selected based on two criteria: (i) the mean ratio of reporter ion intensity originating from salt-treated protein samples (115 and 119) with respect to control protein samples (113 and 117) was more than 1.3 or less than 0.7; and (ii) a *p*-value smaller than 0.05 (Table S3). Based on these criteria, 278 DEPs were identified in soybean leaves, of which 237 (85.3%) displayed increases, and 41 (14.7%) a decrease in abundance under salt stress conditions (Figure 3, Table S4); at the same time, 440 DEPs were identified in soybean roots, of which 354 (80.5%) showed an increase, and 86 (19.5%) showed a decrease in abundance after salt stress treatment (Figure 3, Table S5). Overall, only 50 DEPs were detected in both tissues, of which 9 proteins showed the opposite expression patterns in leaves and roots under salt stress treatments (Figure 3, Tables S4, S5). The results indicated the tissue-specific responses to salt stress at the protein level in soybean leaves and roots. Furthermore, it was obvious that the number of differentially expressed proteins in soybean leaves was less than that obtained from soybean roots, an observation also found in other plant species under salt stress (Liu et al., 2014). The reason for this finding might be attributed to the short period of time that plants were subjected to salt treatment, and the root is the primary site of salinity perception.

### Functional classification of salt-responsive proteins

**Figure 4:**
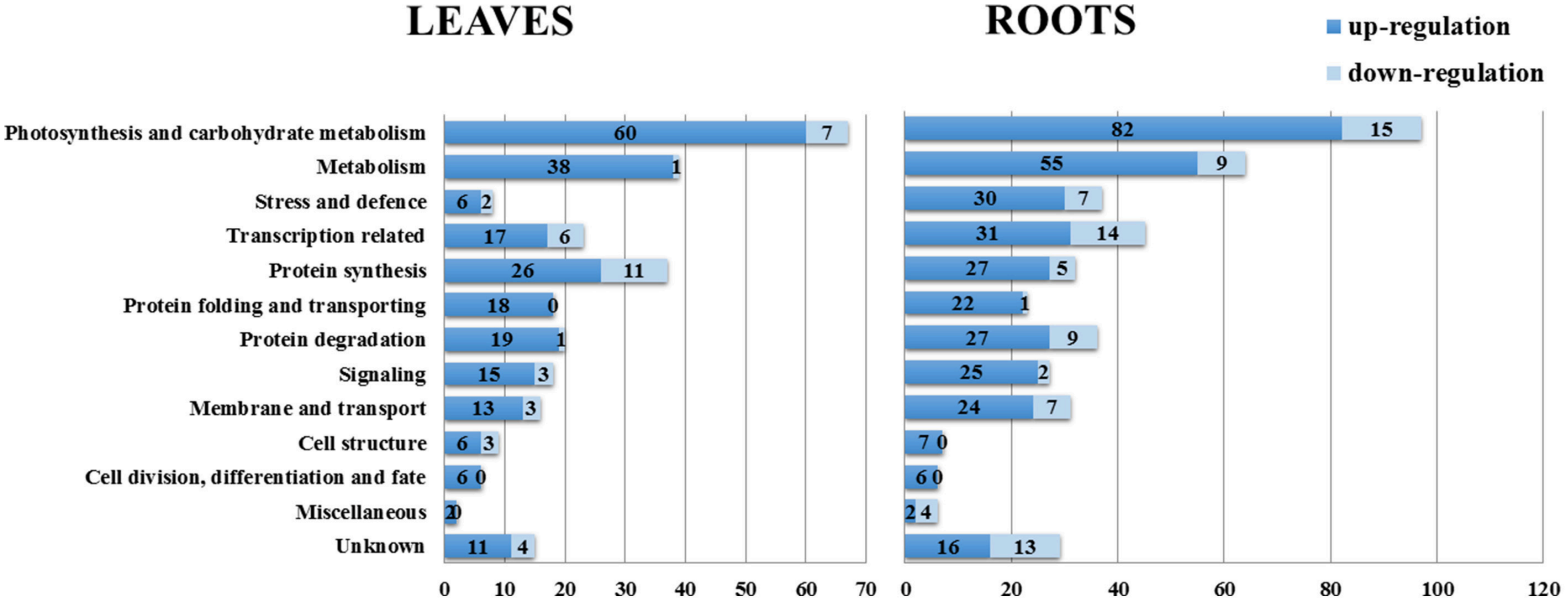
**Distribution of NaCl-responsive proteins in different functional categories**. The plot showed numbers of up-regulation and down-regulation proteins in each functional category in soybean leaves and roots under salt treatment.

On the basis of the BLAST alignment, Gene Ontology, and information from the literature (Zhang et al., 2012; Zhao et al., 2013), all these identified DEPs in leaves and roots were classified into 13 functional categories: photosynthesis and carbohydrate metabolism, metabolism, stress and defense, transcription related, protein synthesis, protein folding and transporting, protein degradation, signaling, membrane and transport, cell structure, cell division/differentiation and fate, miscellaneous and unknown. The distributions of proteins with different functions expressed in the proteome of soybean leaves and roots is illustrated in Figure 4. Our results indicated that the proteins expressed in early salt response were involved in nearly every aspect of plant growth and metabolism.

The most represented DEPs in soybean leaves were associated with photosynthesis and carbohydrate metabolism (24.1%), metabolism (14.4%) and protein synthesis (13.3%); the root DEPs in these first two categories were same as in leaves, while the third category was transcription related (10.2%). In soybean leaves, while there were only 8 DEPs related to stress and defense, whereas the number of DEPs in this category in roots was 37 (Figure 4), and most of which showed increased levels under salt treatment. It has been reported that the early response of soybean to salt stress initially involves the promotion of primary signal perception and transduction, which could be more important than the later responses to salt stress (Pi et al., 2016). In our study, a total of 17 signaling related DEPs in leaves and 27 in roots were identified, including 4 overlapping DEPs in both tissues (Figure 4, Table 1). Moreover, DEPs related to membrane and transport were reported to be involved in early event of salt signal transduction (Luo et al., 2015). In soybean seedlings, 16 proteins in leaves and 31 proteins in roots were identified as being differentially expressed under salt stress (Figure 4, Table 2). Moreover, the numbers of DEPs belonging to the categories of transcription related, metabolism and protein degradation were largely different between the leaves and the roots. These above findings implied the different responses of these biological pathways to short-term salt stress in these two tissues. Detailed information of functional classification of all DEPs in soybean leaves and roots can be found in Tables S4, S5, respectively.

**Table 1:** **Signaling-related proteins differentially expressed under salt stress in soybean leaves and roots**.

	**Leaves**			**Roots**		
	**Protein ID**	**Protein name**	**Mean 115/113**	**Protein ID**	**Protein name**	**Mean 119/117**
(1) Ca^2+^ sensor	UniRef100_C6TNH2	Calcium sensor	4.65	UniRef100_I1K7B7	Calcium ion binding	10.92
	UniRef100_I1K180	Calcium sensing chloroplastic-like	3.31	UniRef100_I1JST7	Calcium ion binding	8.91
	UniRef100_I1K181	Calcium sensing chloroplastic-like	3.27	UniRef100_K7L8B5	Calcium ion binding	4.36
	UniRef100_I1JRM7	Calcium-binding ef hand family protein	0.42			
	UniRef100_A0A762	Calreticulin	0.28			
(2) 14-3-3	UniRef100_E9KNA6	14-3-3 Protein	1.55	UniRef100_I1K9V8	14-3-3 Protein	2.47
	UniRef100_C6TCD3	14-3-3 Protein	1.45	UniRef100_E9KNA6	14-3-3 Protein	2.44
	UniRef100_C6TGW2	14-3-3-like Protein	1.41	UniRef100_C6TGW2	14-3-3-like Protein	2.31
	UniRef100_C6TBV3	14-3-3-like Protein d-like	0.69			
(3) Kinases/phosphatases	UniRef100_I1KIV5	Serine threonine-protein phosphatase 2a 65 kda regulatory subunit a beta isoform-like	2.83	UniRef100_K7LNY2	Serine threonine-protein kinase sepa-like	4.97
	UniRef100_K7MVS5	Protein kinase	1.32	UniRef100_C6TB76	Serine threonine protein phosphatase pp1	3.46
				UniRef100_C6T7X3	Mitogen-activated protein kinase homolog mmk2-like	15.79
(4) Probable receptor				UniRef100_I1LV96	Probable leucine-rich repeat receptor-like serine threonine-protein kinase at3g14840-like	3.55
				UniRef100_K7N5H4	G-type lectin s-receptor-like serine threonine-protein kinase rlk1-like	0.07
(5) Small G protein related	UniRef100_I1KSQ0	RAB GDP Dissociation inhibitor alpha-like	5.28	UniRef100_I1M057	Guanylate-binding family protein isoform 1	6.69
	UniRef100_C6THN1	RAB GDP Dissociation inhibitor alpha-like	5.21	UniRef100_I1L0T1	GTP-Binding protein sar1a	4.61
	UniRef100_C6TJD3	Guanine nucleotide-binding protein subunit beta-like	1.69	UniRef100_K7MNY8	GTPase obg-like	4.10
	UniRef100_I1K5E6	Guanine nucleotide-binding protein subunit beta-like	1.64	UniRef100_I1KX26	Probable ADP-ribosylation factor GTPase-activating protein agd8-like	2.68
				UniRef100_I1K5E6	Guanine nucleotide-binding protein subunit beta-like	2.12
				UniRef100_C6TJD3	Guanine nucleotide-binding protein subunit beta-like	2.11
				UniRef100_K7MYR4	Ras GTPase-activating protein-binding protein	1.30
				UniRef100_C6SY78	Rab6-interacting golgin-like isoform x2	1.35
				UniRef100_C6TB49	Rab6-interacting golgin-like isoform x2	1.31
(6) Phospholipase	UniRef100_I1JI90	Phospholipase c	2.31	UniRef100_I1K784	Phosphatidylinositol 4-kinase alpha	0.08
(7) Other	UniRef100_K7KLE6	Auxilin-like protein	4.61	UniRef100_I1J8S5	Auxilin-related protein 2-like	4.09
	UniRef100_K7K6L6	Auxilin-related protein 2-like	2.13	UniRef100_I1L847	ATP binding	3.34
				UniRef100_K7L5V1	Signal recognition particle 72 kda	2.32
				UniRef100_I1M322	DJ-1 family protein	2.23
				UniRef100_K7MIT8	RJ2 Protein	2.20
				UniRef100_K7LS27	COP1-Interacting protein 4	1.59

**Table 2:** **Membrane and transport-related proteins differentially expressed under salt stress in soybean leaves and roots**.

	**Leaves**			**Roots**		
	**Protein ID**	**Protein name**	**Mean 115/113**	**Protein ID**	**Protein name**	**Mean 119/117**
(1) ABC transporter	UniRef100_Q8W1S2	ABC Transporter f family member 3-like	8.80	UniRef100_Q8W1S2	ABC Transporter f family member 3-like	0.43
				UniRef100_I1NEA8	ABC Transporter f family member 3-like	0.05
				UniRef100_K7M3S0	ABC Transporter g family member 36-like	0.03
(2) Other transporter	UniRef100_C6TJX8	29 kda Chloroplastic-like	7.14	UniRef100_I1LNP0	Cytosolic factor	8.95
	UniRef100_I1KYL0	Web family protein at5g55860-like	3.17	UniRef100_C6TBK9	ER Membrane protein complex subunit 2-like	6.17
	UniRef100_I1KDU7	Proton pump-interactor 1-like	2.82	UniRef100_I1KWA0	Cytosolic factor	4.32
	UniRef100_I1MGI7	Importin subunit alpha-1-like	1.58	UniRef100_C6TDM9	Membrane-associated 30 kda chloroplastic-like	4.22
	UniRef100_I1L0U6	Importin subunit alpha-1-like	1.53	UniRef100_I1KFG3	Outer envelope protein chloroplastic-like	4.02
	UniRef100_C6TL60	Probable plastid-lipid-associated protein chloroplastic-like	0.67	UniRef100_C6TH54	Probable plastid-lipid-associated protein chloroplastic-like	3.31
				UniRef100_I1KYL0	Web family protein at5g55860-like	2.43
				UniRef100_I1LB65	Importin subunit alpha-1-like	1.32
(3) ATPase				UniRef100_I1KPH2	V-type proton ATPase subunit e-like	7.42
				UniRef100_I1KG34	V-Type proton ATPase catalytic subunit a-like	2.23
				UniRef100_I1KVU0	V-Type proton ATPase catalytic subunit a-like	2.17
				UniRef100_D7EYG6	V-Type proton ATPase catalytic subunit a-like	2.17
(4) Ion channel	UniRef100_K7L7I9	Potassium channel	1.40	UniRef100_I1JFN3	Copper transport protein atox1	1.55
	UniRef100_I1JH50	k(+) h(+) Antiporter	0.65			
(5) Vesicle trafficking	UniRef100_I1MZ13	Clathrin heavy chain 1-like	2.42	UniRef100_I1JKB3	Remorin family protein	10.42
	UniRef100_Q39834	Clathrin heavy chain 1-like	2.27	UniRef100_I1K8U2	Clathrin assembly protein at2g25430-like	3.89
	UniRef100_I1LNP0	Cytosolic factor	1.80	UniRef100_Q39834	Clathrin heavy chain 1-like	2.65
	UniRef100_C6TJF6	Annexin-like protein	1.52	UniRef100_I1JIA0	Clathrin heavy chain 1-like	1.88
	UniRef100_K7LQG2	Aquaporin pip2-7	1.31	UniRef100_I1MZ13	Clathrin heavy chain 1-like	1.83
	UniRef100_I1M8Y5	Clathrin interactor epsin 1-like	0.66	UniRef100_I1N727	Nuclear pore membrane glycoprotein 210-like	1.39
				UniRef100_I1M7W9	Aquaporin protein pip11	0.07
				UniRef100_K7M2D8	Trafficking protein particle complex subunit 10-like	0.03
				UniRef100_C6TMQ6	Stomatin-like protein	13.32
(6) Other				UniRef100_C6TKQ1	Gem-like protein 1-like	11.09
				UniRef100_I1N0D7	Protein to RNADo 1-like	9.53
				UniRef100_K7KMV4	Endonuclease or glycosyl hydrolase	3.74
				UniRef100_I1KIU7	Protein CASP-like	1.38
				UniRef100_I1JV52	Gem-like protein 1-like	0.59
				UniRef100_I1LJG0	Nucleotide-diphospho-sugar transferase family protein	0.02

### Protein-protein interaction among DEPs

**Figure 5:**
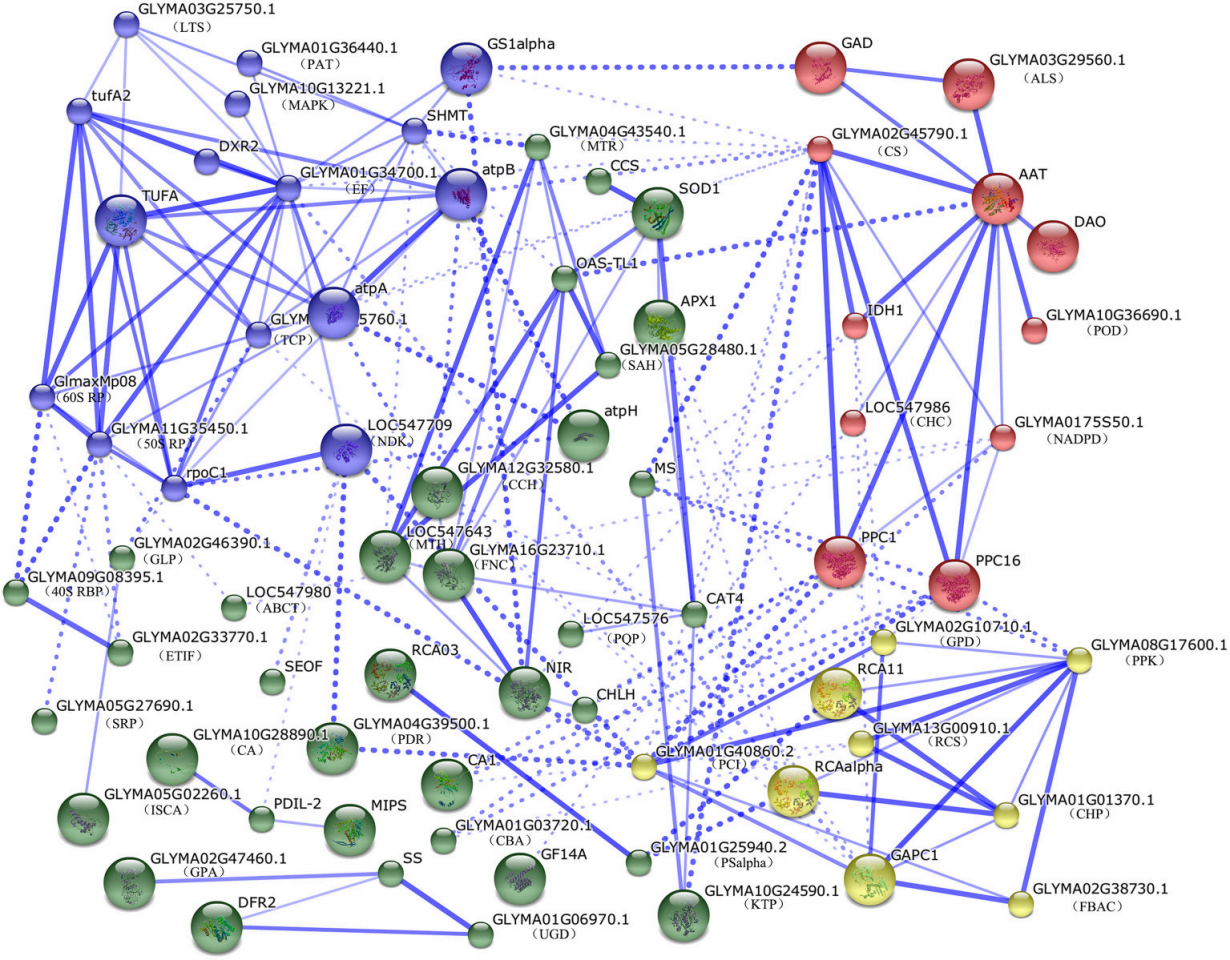
**The protein-protein interaction (PPI) network of DEPs in soybean leaves and roots based on STRING analysis**. A total of 104 differentially abundant proteins represented by 72 unique proteins from soybean are shown in PPI network. Nodes in different colors belong to four main groups. Stronger associations are represented by thicker lines. Detailed information on protein names and abbreviations can be found in Table S4.

To predict the relationship among all these identified DEPs in soybean leaves and roots, a protein-protein interaction (PPI) networks were generated using the web-tool STRING 9.1. A total of 104 differentially abundant proteins represented by 72 unique proteins from soybean were shown in the PPI network (Figure 5, Table S6) based on the published literature and other experimental evidence (Zhao et al., 2015). Four functional modules forming tightly-connected clusters were illuminated in the network (Figure 5). Nodes in different colors belong to four main groups. Stronger associations are represented by thicker lines. In Module 1 (blue nodes), six protein synthesis related proteins, five amino acid metabolism related proteins, two ATP synthases, a TCP as well as a MAPK appeared closely linked. This implied that amino acid metabolism, protein synthesis and energy supply were active and cooperated closely in soybean seedlings under salt stress. Module 2 (red nodes) included multiple enzymes involved in the TCA cycle, glycolysis, fatty acid biosynthesis and nitrogen metabolism. These linked proteins indicated that a synergistic system for carbon and nitrogen metabolism may play important roles in salt response. Moreover, PPC1 and PPC16, two key enzymes in organic acid metabolism were linked with module 3 (yellow nodes), which included ten proteins functioning in photosynthesis, carbohydrate and energy metabolism. Furthermore, proteins involved in protein folding, transporting, ROS scavenging, as well as some signaling components were assigned in Model 4 (green nodes). This indicated that proteins in this network played important functions in redox homeostasis, response to stress, signal transduction and protein metabolism.

### Regulation of some salt-responsive proteins at the mRNA level

**Figure 6:**
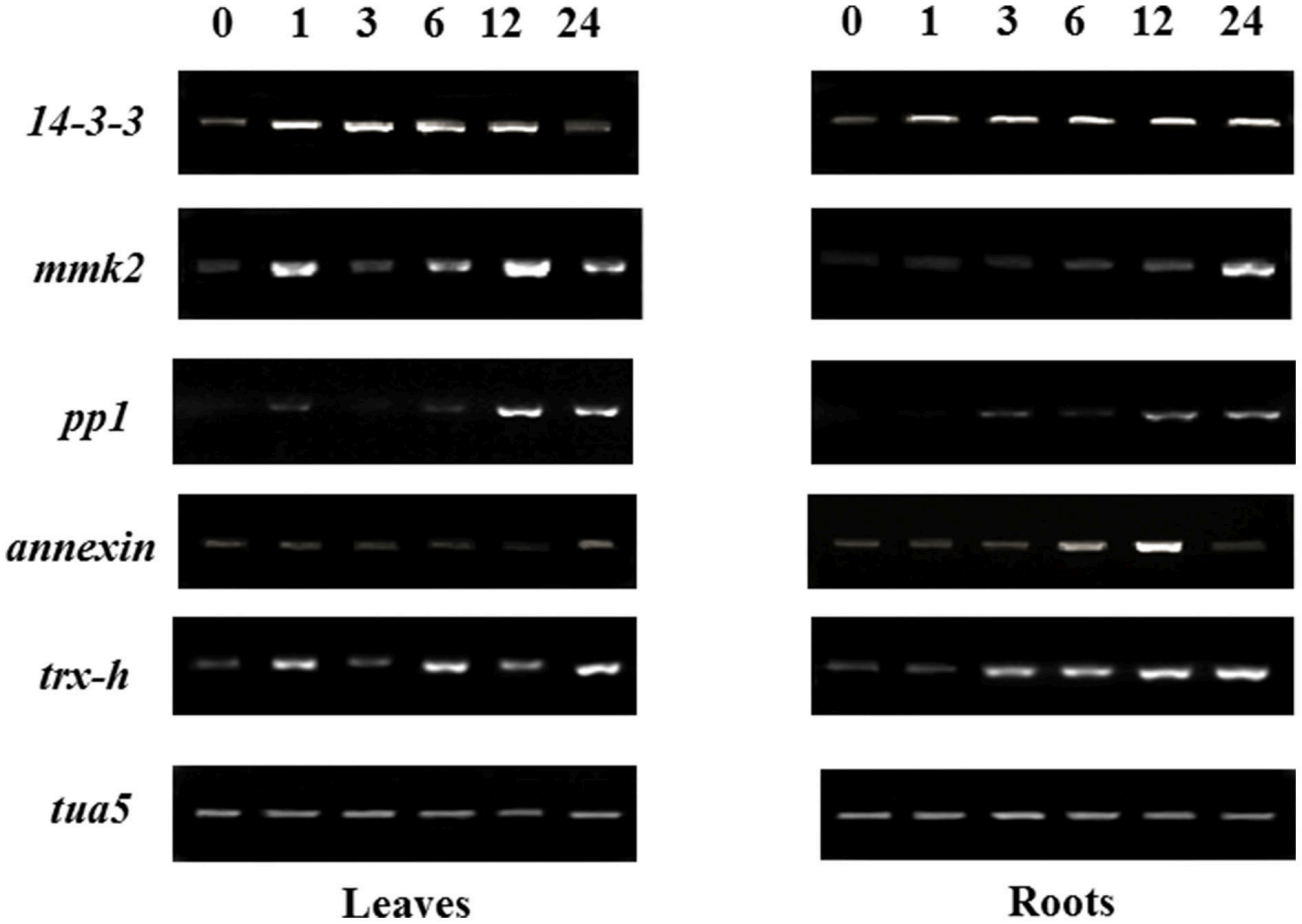
**Gene expression analysis of salt responsive proteins identified from the iTRAQ assay**. After treated soybean seedlings with 200 mM NaCl for 0 (control), 1, 3, 6, 12, 24 h, leaves and roots were harvested for semi-quantitative RT-PCR, and *tua5* gene was used as internal RNA reference in leaves and roots.

In order to further understand the correspondence between proteins and their mRNA expression patterns, transcriptional analyses of five randomly selected proteins showing significant changes under salt stress were studied by semi-quantitative RT-PCR (Figure 6). After salt treatment, the changes of the mRNA levels in four genes correlated with changes at the protein levels as indicated by iTRAQ analysis, this included a *14-3-3, mmk2, pp1* and *trx-h*. The mRNA of *annexin* showed a down-regulated trend in soybean leaves when treated for 12 h, however, annexin had a higher protein expression level (Table 2). In contrast, the *annexin* gene in soybean roots exhibited constant up-regulation during the first 12 h of salt treatment, and then was down-regulated at 24 h after salt treatment, which showed no different expression according to our proteomic analysis. The mRNA levels of *annexin* gene showed poor agreement with corresponding protein expression levels, probably resulting from protein post-transcriptional regulation and modulation of its binding to various ligands under salt stress (Vedeler et al., 2012).

## Discussion

To cope with salt stress, soybean plants have evolved complex salt-responsive signaling and metabolic processes at the cellular, organ, and whole-plant levels. In our study, morphological and physiological changes in soybean seedlings were observed which represented the plant's early response to salt stress (Luo et al., 2015). The present study involved a comparative analysis of early salt stress responses to leaf and root of soybean seedlings using quantitative proteomic approach. Among 6610 identified proteins, a total of 278 proteins in leaves and 440 proteins in roots in responded to NaCl stress treatments. The functions of these salt responsive proteins and their main pathways are discussed further below.

### Signal transduction-associated proteins

Understanding salt-responsive signaling pathways is currently a hot topic in research of plant salt stress. Using an iTRAQ approach, a total of 37 signaling-related proteins were identified in this present study, and a majority of them exhibited tissue-specific expression (Table 1). Based on the roles of these proteins in signal perception and transduction pathways, we classified these proteins into several groups, including Ca^2+^ sensors, 14-3-3s, kinase and phosphatases, probable receptors and small GTP binding proteins (Table 1).

Ca^2+^ plays a vital role as second messenger in plant cells in response to environmental stimuli. Plants have evolved a diversity of proteins function as Ca^2+^ sensors that facilitate their regulation of target proteins and thereby coordinate various signaling pathways by binding Ca^2+^ using the evolutionarily conserved EF-hand motif (DeFalco et al., 2010). In our study, calcium sensor, calcium sensing chloroplast-like proteins and three calcium ion binding proteins were up-regulated more than threefold under salt stress (Table 1). The EF-Hand containing protein has a single EF-hand motif, which is used as a molecular device to recognize specific Ca^2+^ signals through binding Ca^2+^ to change its conformation to interact with down-stream proteins (Ma et al., 2014). A previous report has shown that the EF-hand calcium binding protein decreased its abundance at the stress time point of 72 h, but was found to be significantly up-regulated at the stress time point of 144 h in soybean roots (Ma et al., 2014). In our study, this protein was down-regulated 0.4-fold in soybean leaves under salt stress. Consistent with a previous study in rice leaves under osmotic stress (Zang and Komatsu, 2007), the abundance of calreticulin, a calcium-binding chaperone protein that plays a pivotal role in regulating calcium homeostasis and protein folding in the endoplasmic reticulum, was decreased in soybean leaves under salt stress. These findings indicated that signaling pathway mediated by calcium is an important strategy of soybean seedlings in coping with salt stress.

14-3-3 proteins were reported to regulate the activities of many proteins involved in signal transduction and play important roles in stress responses in higher plants (Roberts et al., 2002). In the current study, four 14-3-3 proteins in leaves and three 14-3-3 proteins in roots with two proteins in common were detected in both tissues, most of which showed dramatically up-regulated expression at translational levels (Table 1). Furthermore, semi-quantitative RT-PCR results showed that *14-3-3* transcript level increased significantly after salt treatment in both tissues, which expressed to the highest level either at 12 or 24 h after salt stress (Figure 5). Consistent with our results, a 14-3-3 protein was also up-regulated in *Brachypodium distachyon* leaves under salt stress (Lv et al., 2014), and the transcription of 14-3-3 genes in cotton showed an increasing pattern under salt stress (Sun et al., 2011). In addition, several analyses have shown that 14-3-3 proteins can be phosphorylated and interact with many proteins in various functional groups to play roles in signal pathways under salt stress (Lv et al., 2014; Zhou et al., 2014).

In the present study, three protein kinases and two protein phosphatases were identified, which showed enhanced expression levels under salt stress (Table 1). Specifically, mitogen-activated protein kinase homolog mmk2-like was up-regulated more than 15-fold under salt stress. The MAPK family is reported to play various roles in intra- and extra-cellular signaling in plants by transferring the information from sensors to responsers, which act as points of convergence in abiotic stress signaling (Sinha et al., 2011). The serine threonine protein phosphatase PP1 also showed enhanced expression level under salt stress in soybean roots. Although there is no direct evidence in support of the significant role of PP1 in plant salt tolerance, the PP1 regulatory protein RICE SALT SENSITIVE 1 (RSS1) was identified recently through a combined approach of genetic screening for salt tolerance in rice and yeast two-hybrid screening, and the loss of RSS1 results in short root and dwarf phenotypes under high salt (Ogawa et al., 2011). Similar to the expression patterns at the translational level, up-regulation of MMK2 and PP1 were also validated by semi-quantitative RT-PCR analysis, which indicated that these proteins were regulated at the transcriptional level as well (Figure 6).

In addition, several GTP-binding proteins and GTPase-activating proteins, known to be involved in controlling the transmission of extracellular signals to intracellular pathways exhibited increased expression levels under salt stress, suggesting that G-protein-coupled receptors were dynamically regulated to cope with salinity in soybean leaves. These results indicated that signal perception and transduction had been highly enhanced at early stages of plant stress response, thereby improving the activities of stress-responsive pathways in the leaves and roots of soybean plants exposed to salt stress.

### Membrane and transport-related proteins

Membrane proteins fulfill critical functions in the transport of ions and organic molecules, which also play important roles in ion homeostasis. Under salinity conditions, Na^+^/K^+^ ratios and Na^+^ concentration increase in soybean roots and leaves causing hyper osmotic stress, cellular ionic toxicity and oxidative stress (Ma et al., 2014). ABC transporters are known to transport stress-related secondary metabolites, such as alkaloids, terpenoids, polyphenols and quinines to protect plants against salt stress (Yazaki, 2006). The present proteome analysis indicated that the ABC transporter f family member 3-like was up-regulated in soybean leaves under salt treatment (Table 2). Increased expression level of an ABC transporter was also found in cotton seedlings suggesting that it may play an important role in salt stress responses (Li et al., 2015). However, three other ABC transporter f family members were decreased in soybean roots under salt stress (Table 2). The expression differences implies that different gene family members probably have diverse functions in different tissues to cope with various stresses. The importin subunit alpha-1-like proteins were identified with increased expression levels in both tissues under salt stress (Table 2). Importin α is well known as an adaptor that functions with importin β in the nuclear import of proteins containing specific nuclear localization signals (NLSs) which was reported to be regulated by phosphorylation (Hachet et al., 2004). By employing a genetic screen in *Arabidopsis*, an importin β-domain/karyopherin protein was identified to be involved in nucleocytoplasmic trafficking under cold, osmotic stress and ABA treatments (Chinnusamy et al., 2007). However, the detailed function of this protein in plant salt response is not clear and deserve further studies.

A total of four V-type proton ATPase were up-regulated in soybean roots (Table 2). H^+^-ATPase plays an essential role in the maintenance of ion homeostasis in plant cells, which was identified as an important salt stress marker protein according to several proteomic studies (Kerkeb et al., 2001; Jiang et al., 2007; Li et al., 2015; Luo et al., 2015). Thus, increased activities of these enzymes may be an effective strategy for osmotic adjustment, which reduces the Na^+^ concentration in the cytosol of plants under salt stress.

Finally, we identified 15 vesicle trafficking-related proteins that exhibited differential expression patterns in soybean seedlings under salt stress (Table 2). Aquaporin (AQP) proteins function in transporting water and other small neutral solutes or gasses through the biological membranes, which is crucial for plants to survive in drought or salt stress conditions (Sade et al., 2010). AQP contains two subfamilies, the plasma membrane intrinsic proteins (PIPs) and the tonoplast intrinsic proteins (TIPs), which are most abundant in the plasma membrane and vacuolar membrane, respectively. Many genes encoding PIPs have been identified from different plant species, and overexpression of these genes have been reported to enhance plants salt tolerance (Sade et al., 2010; Hu et al., 2012; Liu et al., 2013; Xu et al., 2014; Sreedharan et al., 2015). Here, two isoforms of PIP showed different expression patterns in soybean leaves and roots in response to salt stress (Table 2). This may be attributed to tissue- and time-specific expression manners of different PIP isoforms under salt stress. In addition, an annexin-like protein was up-regulated by salt stimulus in soybean leaves in this study. Annexin functions as a Ca^2+^-permeable channel in the plasma membrane to form a ROS-stimulated passive Ca^2+^ transport pathway (Laohavisit et al., 2010), which was reported to be induced by salinity in variety of plant species based on proteomics data (Lee et al., 2004; Manaa et al., 2013), indicating the significance of this protein in plant salt stress tolerance. In summary, the identified membrane and transport related proteins were consistent with the physiological processes of maintaining ion homeostasis and membrane stability, which are crucial for plant growth during salt stress.

### Stress-related proteins

Salt stress causes the overproduction of reactive oxygen species (ROS), which oxidizes proteins, lipids, carbohydrates and DNA and irreversibly damages plant cells (Gill and Tuteja, 2010). The antioxidant properties of plant cells is usually represented by the general radical scavenging capacities of peroxidases (POD), ascorbate peroxidase (APX), glutathione S-transferase (GST) and superoxide dismutase (SOD). In the present study, the increase of POD, APX, GST and SOD abundances in soybean roots was outlined (Table S5), which is similar to other salt-responsive species (Jiang et al., 2007; Peng et al., 2009; Du et al., 2010). These results were confirmed by the activities of ROS-scavenging enzymes SOD and POD in soybean leaves and roots under 200 mM NaCl treatment for the initial 12 h (Figures 2C–F). Thus, the expression changes of these proteins under salt stress implied that the antioxidative defense system in soybean seedlings was provoked by salt treatment.

In addition to the redox related proteins, plants have developed cross-tolerance mechanisms to cope with different stresses (Zhang et al., 2012). From our iTRAQ data, some biotic stress-related proteins were induced under salt stress conditions, such as disease resistance protein rpp13 and pathogenesis-related protein class 10 (PR10), which mediates tolerance to heavy metals (Wang Z. Q. et al., 2014) and pathogen attack (Coumans et al., 2009). Interestingly, several major latex proteins (MLPs) were up-regulated in soybean roots but down-regulated in soybean leaves under salt stress (Tables S2, S3), which was in agreement with the proteomic findings of soybean root tips under flooding (Yin X. et al., 2014). MLPs were found only in plants and associated with fruit and flower development and in pathogen defense responses. So far, only one study reported that transcription of the *mlp* gene in cotton was rapidly induced by NaCl and overexpression of this gene enhanced salt tolerance in *Arabidopsis* (Chen and Dai, 2010). However, the specific biological function of MLP and whether up-regulation of MLP correlates with enhanced salt tolerance in soybean plants are unknown and to the best of our knowledge, it may represent a novel salt-stress-responsive protein in soybean plants. Furthermore, some drought stress-related proteins, e.g., dehydrin and desiccation protectant protein lea14 homolog, also responded to salt stress in our study (Tables S4, S5). These proteins provide novel insights into the understanding of the cross-tolerance mechanisms in soybean seedlings in response to biotic and abiotic stresses.

### Metabolisms

A large number of DEPs were found to be involved in nitrogen and amino acid metabolism. Glutamine synthetase, the key enzyme in plant NH^4+^ metabolism, has been reported to play an important role in enhancing rice tolerance to salt and chilling stresses (Hoshida et al., 2000). We also found it up-regulated in soybean leaves under salt stress. Cysteine synthase is responsible for the final step in cysteine biosynthesis, the key limiting step in producing glutathione (GSH), which is involved in resistance to adverse stresses. Liu et al. (Liu et al., 2014) used comparative proteomic methods to show that cysteine synthase was induced in a salt-tolerant rice cultivar but down regulated in salt-sensitive rice leaves. In this study, four cysteine synthases were identified to be up-regulated in soybean roots (Table S5), and appeared tightly linked with other proteins in the soybean PPI network (Figure 5). Aspartate aminotransferase (AAT) catalyzes the conversion of α-ketoglutarate and aspartate to glutamate and oxaloacetate (Hodges, 2002). Proteomic analysis has found the salt also induced AAT in rice roots (Nam et al., 2012), which is consistent with the results in this study. There were many other salt-responsive proteins related to amino acid metabolism identified only in soybean roots, such as aminotransferase-like, glutamate decarboxylase, alanine aminotransferase, serine hydroxymethyltransferase, phosphoserine aminotransferase, asparagine synthetase and serine hydroxymethyltransferase (Table S5). These results indicated that amino acid and nitrogen metabolism were enhanced in soybean seedling leaves and roots under salt stress. Significantly, 14 lipoxygenases showed enhanced levels both in soybean leaves and roots under salt stress, which suggested that lipid metabolism changes under salt stress and may play important roles in soybean seedling growth.

Salt adaptation of plants requires complex rearrangements of metabolism with interactions between several metabolic pathways. Furthermore, one of the most striking observations in our study was the increase of several cytoplasmic enzymes engaged in secondary metabolism in roots under short-term salt stress. Dihydroflavonol reductase (DFR), a key enzyme involved in anthocyanin biosynthesis and proanthocyanidin synthesis, was reported to be up-regulated by salt stress in soybean seedlings (Ma et al., 2012), which was consistent with results in our study (Tables S4, S5). Caffeic acid 3-O-methyltransferase was up-regulated more than 14-fold in soybean leaves under salt stress (Table S5). This protein is involved in lignin biosynthesis. The accumulation of this enzyme under salt stress could be related to increased lignification of the cell wall—a modification to avoid water loss induced by osmotic stress (Simova-Stoilova et al., 2015), a process that was reported to be up-regulated by drought stress in soybeans (Alam et al., 2010). Interestingly, several enzymes related to flavonoid compounds metabolism were identified in our study. Recent evidence has suggested that salinity stress strengthened the accumulation of flavonoids, which could play vital roles downstream of soybean tolerance to salt stress (Qu et al., 2016). Isoflavone reductase catalyzes the reduction of 2′-hydroxyformononetin to vestitone, which is the penultimate step in the synthesis of medicarpin in the general flavonoid biosynthesis pathway. In the present study, isoflavone reductase homologs were induced in soybean roots under salt stress (Table S5), which was inconsistent with previous results (Sobhanian et al., 2010). Moreover, increased expression levels of three chalcone isomerase were identified in soybean roots (Table S5). This result supported the significant correlations of chalcone metabolic enzymes in soybean's tolerance to salinity (Pi et al., 2016). These results suggested that the biosynthesis of these secondary metabolites was associated with salt stress response in soybean seedlings.

## Conclusions

In the present study, mophological and physiological changes were determined in soybean leaves and roots treated with 200 mM NaCl for up to 48 h, and the results supported the treatment time of 12 h for a proteomics survey. An iTRAQ-based proteomic technique was employed to compare the abundance of proteins in untreated and 200 mM NaCl treated soybean leaves and roots for 12 h. In total, 278 and 440 differentially changed proteins in the leaves and in the roots, respectively, which were classified into 13 categories. As a result, we gained new information about proteins in soybean seedlings and their roles in the salt stress response. First, stress signal transduction and membrane proteins in soybean were activated at the early stages of salt stress treatment. The second strategy involved the upregulation of proteins leading to ROS scavenging and cross-tolerance to other biotic and abiotic stresses. Third, rearrangements of several metabolic pathways led to salt adaptation of soybean seedlings. Protein-protein interaction analysis implicated that protein metabolism, energy supply and photosynthesis collectively functioned to re-establish cellular homeostasis under salt stress. Furthermore, semi-quantitative RT-PCR results suggested that the expression of some proteins (e.g., *annexin*) could be regulated post-transcriptionally. These results may contribute to the existing knowledge on the complexity of soybean protein changes that occur in response to salt stress. Further studies for gene function analysis are needed to further clarify the molecular mechanism under salt stress in soybean roots and leaves.

## Author contributions

WJ: method optimization, data analysis, drafting the manuscript. RC: data analysis and semi-qRT-PCR analysis. SL and RL: physiological analysis, protein isolation and data analysis. ZQ: manuscript preparation. YL and XZ: seedlings treatment and physiological analysis. SC: overall design of the experiments, and manuscript preparation. JL: overall design of the project and experiments, and manuscript preparation.

### Conflict of interest statement

The authors declare that the research was conducted in the absence of any commercial or financial relationships that could be construed as a potential conflict of interest.
